# The common transcriptional subnetworks of the grape berry skin in the late stages of ripening

**DOI:** 10.1186/s12870-017-1043-1

**Published:** 2017-05-30

**Authors:** Ryan Ghan, Juli Petereit, Richard L. Tillett, Karen A. Schlauch, David Toubiana, Aaron Fait, Grant R. Cramer

**Affiliations:** 1 Department of Biochemistry and Molecular Biology, University of Nevada, Reno, NV 89557 USA; 2 Nevada INBRE Bioinformatics Core, University of Nevada, Reno, NV 89557 USA; 3 Telekom Innovation, Laboratories and Cyber Security Research Center, Department of Information, Systems Engineering, Ben Gurion University, Beer Sheva, Israel; 4 Ben-Gurion University of the Negev, Jacob Blaustein Institutes for Desert Research, 84990 Midreshet Ben-Gurion, Israel

**Keywords:** Circadian clock, Chromosome organization, Epigenetic modification, Fruit development, Grape berry, Network analysis, RNA-seq, Transcriptomics, *Vitis vinifera* L

## Abstract

**Background:**

Wine grapes are important economically in many countries around the world. Defining the optimum time for grape harvest is a major challenge to the grower and winemaker. Berry skins are an important source of flavor, color and other quality traits in the ripening stage. Senescent-like processes such as chloroplast disorganization and cell death characterize the late ripening stage.

**Results:**

To better understand the molecular and physiological processes involved in the late stages of berry ripening, RNA-seq analysis of the skins of seven wine grape cultivars (Cabernet Franc, Cabernet Sauvignon, Merlot, Pinot Noir, Chardonnay, Sauvignon Blanc and Semillon) was performed. RNA-seq analysis identified approximately 2000 common differentially expressed genes for all seven cultivars across four different berry sugar levels (20 to 26 °Brix). Network analyses, both a posteriori *(*standard) and a priori (gene co-expression network analysis), were used to elucidate transcriptional subnetworks and hub genes associated with traits in the berry skins of the late stages of berry ripening. These independent approaches revealed genes involved in photosynthesis, catabolism, and nucleotide metabolism. The transcript abundance of most photosynthetic genes declined with increasing sugar levels in the berries. The transcript abundance of other processes increased such as nucleic acid metabolism, chromosome organization and lipid catabolism. Weighted gene co-expression network analysis (WGCNA) identified 64 gene modules that were organized into 12 subnetworks of three modules or more and six higher order gene subnetworks. Some gene subnetworks were highly correlated with sugar levels and some subnetworks were highly enriched in the chloroplast and nucleus. The petal R package was utilized independently to construct a true small-world and scale-free complex gene co-expression network model. A subnetwork of 216 genes with the highest connectivity was elucidated, consistent with the module results from WGCNA. Hub genes in these subnetworks were identified including numerous members of the core circadian clock, RNA splicing, proteolysis and chromosome organization. An integrated model was constructed linking light sensing with alternative splicing, chromosome remodeling and the circadian clock.

**Conclusions:**

A common set of differentially expressed genes and gene subnetworks from seven different cultivars were examined in the skin of the late stages of grapevine berry ripening. A densely connected gene subnetwork was elucidated involving a complex interaction of berry senescent processes (autophagy), catabolism, the circadian clock, RNA splicing, proteolysis and epigenetic regulation. Hypotheses were induced from these data sets involving sugar accumulation, light, autophagy, epigenetic regulation, and fruit development. This work provides a better understanding of berry development and the transcriptional processes involved in the late stages of ripening.

**Electronic supplementary material:**

The online version of this article (doi:10.1186/s12870-017-1043-1) contains supplementary material, which is available to authorized users.

## Background

Fruits are specialized organs that encapsulate seeds. Botanically a fruit is the ripened ovary or carpel of a flower. Functionally it allows the development of and is a vehicle for the dispersal of seeds. There are different kinds of fruits. Fleshy fruit examples are grapes, apples and oranges; dry fruits include cereal grains and nuts. Both fleshy and dry fruits have similar regulatory subnetworks and developmental programs [1].

Grape berries are formed on flower clusters or inflorescences. Grape berry development and ripening involve complex physical and molecular changes [2], including color development, softening, volatile production, acid catabolism, and sugar accumulation. These processes at maturity or peak ripeness produce attractive signals for human, avian and other vectors of seed dispersal. Grape berries have many bioactive compounds, like polyphenols and carotenoids with bright colors and aromas that signal their edibility and health-related benefits [3, 4].

Grape berry color change and sugar accumulation are common metrics for ripeness. These processes change significantly at veraison, the start of the ripening stage in grapes. Sugar accumulation can vary with genotype and environment [2]. For example, higher temperatures lead to higher sugar accumulation in the berry at the optimum time of ripeness (as defined by the winemaker). Many times the winemaker will taste the berries to determine optimum flavor and time to harvest.

Nonetheless, sugar accumulation is a wine industry standard for assessing grape maturity. Wine grapes are typically harvested between 22 and 25 °Brix. Other measures of fruit maturity exist (e.g. total acidity or the sugar to total acidity ratio), but the sugar level remains a simple and standard measurement of grape maturity in the wine industry. Measurement of sugar levels in grapes is an easy and accurate measurement; all that is needed is a simple refractometer that measures soluble solids (°Brix), which are essentially made up of glucose and fructose sugars. Wine grape berries typically can reach 25% sugar if allowed to ripen fully. Higher concentrations can be achieved, but this is often due to dehydration of the berry. A recent paper characterizes in detail the effects of postharvest dehydration in these late stages of berry ripening [5], which are beyond the stages studied in the present study.

Sugars affect plant growth and development. Sugars provide energy for cellular respiration and transcriptionally signal and regulate gene activity, allowing the fine-tuning of fruit metabolism and development [6, 7]. Sugar can interact with hormones and the circadian clock to regulate gene expression [8]. In addition, sugar can induce senescence [9] and inhibit the expression of photosynthetic genes [10]. Some of the sugar sensors have been identified in plants [8] including hexose kinase (HXK1), SNF1 related protein kinase 1.1 (SnRK1.1) and target of rapamycin (TOR). A glucose phosphate transporter (GPT2) appears to be an important sugar sensor in the chloroplast [10].

Sugars can influence fruit development as well [11–13]. Sugar signaling has been linked to wheat grain development [13]. In strawberry, tomato [12], and grape [14], sugar influences the expression of the ABA-stress-ripening (*ASR*) gene, a transcription factor, which when it is over-expressed or silenced, accelerates or delays fruit ripening, respectively [12]. In grapes, sugars enhance anthocyanin development and the expression of many genes, including the sugar sensor, *HKT1* (High-affinity K (potassium) Transporter 1) [11, 15, 16].

Other factors can affect fruit ripening, such as epigenetic regulation and hormones [1, 2]. An investigation of the tomato methylome showed epigenetic control of ripening that was tissue and developmentally specific at the breaker stage [17] when color begins to develop. Grape berry development is likely to be under epigenetic regulation as well [18, 19].

Hormonal regulation of fruit development is perhaps best studied in tomato [1]. A complex interplay of hormones are involved at different stages of fruit development. A number of grape studies indicate that different aspects of ripening are also under hormonal control by auxin, ethylene, abscisic acid and other hormones [2]. Most of these grape studies were focused on veraison.

A few studies have focused on the late ripening stages of grapes [2, 5, 20–22]. This stage of development represents a senescence-like phase preparing the fruit for seed dispersal with several degradative processes occurring including chloroplast disintegration [23, 24] and cell death [22]. A large number of genes and physiological processes appears to be operating including genes involved with ethylene signaling and flavor pathways [20] in the skin of Cabernet Sauvignon berries in the late stages of ripening over a range of °Brix levels (22 to 37 °Brix). This present study focuses on a better definition of a core set of late ripening genes by expanding upon our previous findings in Cabernet Sauvignon. To better understand fruit ripening processes in this stage, four red-skinned and three white-skinned grape cultivars were studied: Cabernet Franc, Cabernet Sauvignon, Merlot, Pinot Noir, Chardonnay, Sauvignon Blanc and Semillon, respectively. A narrower range of °Brix levels was selected to restrict the set of candidate genes involved in berry skins to those genes that are expressed around the optimal sugar levels that flavor maturity develops; berry skins are the primary source of aroma, flavor and color in grapes [25].

Transcriptomic and gene network analyses can be used to infer active physiological processes in organisms. To derive and understand complex gene subnetworks (modules) in cells or whole organisms, scientists use gene co-expression network analysis approaches to identify highly connected gene subnetworks with highly enriched gene ontology (GO) categories [26–29]. WGCNA (Weighted Gene Co-expression Network Analysis) is one approach that was effectively used to identify highly connected hubs in gene subnetworks for *Arabidopsis* [30, 31] and *Vitis* [32]. Another approach, petal [29], was developed recently to provide a truly scale-free and small-world network model for large-scale Omics analyses. A common set of transcriptional changes occurring in the late ripening stages for all cultivars was identified in this study using a standard (a posteriori) approach of gene mapping to known biochemical pathways and gene network (a priori) approaches. These approaches have elucidated multiple transcriptional processes of grape berry ripening at the mature stage and identified top hub genes in these gene subnetworks some of which involve autophagy, photosynthesis, chromosome organization and the circadian clock.

## Results

Throughout September and October of 2012, whole berry clusters were harvested at the Valley Road Nevada Agricultural Experiment Station Experimental Vineyard. Seven grape cultivars were harvested: Cabernet Franc, Cabernet Sauvignon, Merlot, Pinot Noir, Chardonnay, Sauvignon Blanc and Semillon. Individual berry skins were separated immediately from the whole berry and the individual sugar (°Brix) level of the berries was determined. RNA-seq profiling of transcript abundance during the late stages of development was then conducted on berry skins at different sugar levels (see Methods for details). Prior to signal filtering, there were 27,926 expressed genes out of 29,971 annotated genes in the V1 reference genome (Additional file 1). Independent filtering of lowly expressed genes by minimum counts mapped (see Methods) reduced the count to 16,606 genes for downstream analysis (Additional file 2).

**Fig. 1:**
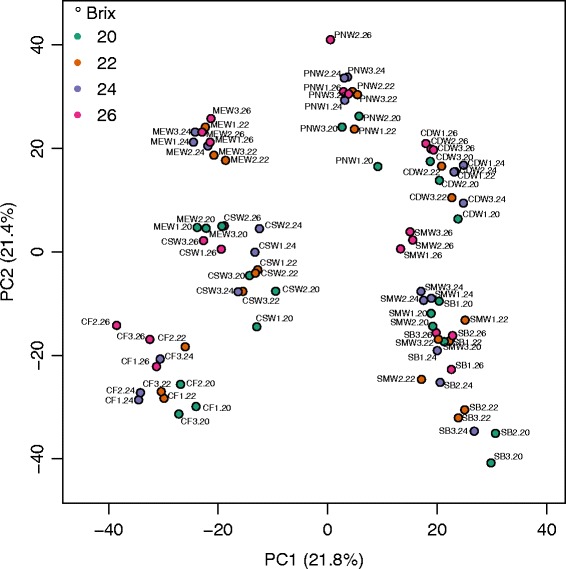
A PCA plot of berry skin samples according to their normalized counts per million. The first (*PC1*) and second (*PC2*) components are represented. Samples corresponding to three biological replicates from four °Brix levels were analyzed. °Brix levels are colored across cultivars. Sample abbreviations represent the cultivar, replicate number and Brix level, respectively. Cultivar abbreviations are Cabernet Franc (*CF*), Sauvignon Blanc (*SB*), Cabernet Sauvignon (*CS*), Merlot (*ME*), Pinot Noir (*PN*), Chardonnay (*CD*) and Semillon (*SM*)

A principal components analysis (PCA) was performed (Fig. 1) to validate sample uniformity and investigate the degree of separation between cultivar and °Brix effects. Cultivars were distinctly separated on the 1st principal component explaining 21.8% of the variance, with red and white cultivars segregating together and away from one another. °Brix levels segregated along the 2nd principal component explaining 21.4% of the variance, in some cases distinctly from one another (e.g. Merlot at 20 °Brix, Semillon and Chardonnay both at 26 °Brix). The cultivars separated in a similar pattern as in a previous study [33], with Pinot Noir again segregating between red and white cultivars.

While there were many transcripts changing that were different for different cultivars, in this presentation of the results we focus on the common changes in transcript abundance amongst the seven grape cultivars at different °Brix levels. We define a common differentially expressed gene (DEG) as the gene’s transcript abundance changing significantly in at least one Brix comparison of all possible comparisons (e.g. 24 vs 22, 26 vs 20, 24 vs 22, etc.) AND this had to occur for at least one comparison in every cultivar. We identified 2108 common differentially expressed genes (DEGs) using a series of contrasts between each °Brix level (Additional files 3 and 4).

In the following analyses, we utilized the DEGs to conduct a “standard” transcriptomic analysis approach, followed by two different gene co-expression network analysis approaches. In the first approach (standard network analysis), gene set enrichment of gene ontologies (GO) was utilized to identify “genes of interest”. They were then mapped to known physiological and biochemical pathways (a posteriori approach) to gain further insight. In the second approach, we applied two different gene co-expression network analyses, WGCNA and petal, to the set of 16,606 filtered and quality-controlled transcripts (a priori approach) and evaluated some of the gene subnetworks and hubs identified.

### Standard transcriptomic analysis

#### Gene ontology enrichment analysis

Enriched GO categories for the 2108 DEGs for the common genes of all cultivars (the °Brix effect) were identified (Additional file 5) and were depicted in a GO network (Additional file 6). The color and size of the circles signify the level of enrichment and the number of genes in each set, respectively. Approximately 400 GO categories were overrepresented after correcting for multiple hypothesis testing (Additional file 5). Mapping the DEGs on the cellular overview of the Vitiscyc [34] webpage showed that these transcripts were widespread across most biochemical pathways (data not shown). These results indicated that grape berry ripening in the late stages was broad and complex across many biochemical pathways.

Some of the top overrepresented GO categories included membrane, regulation of hormone levels, catabolic process and response to abiotic stress. Other interesting GO categories included developmental process, response to light, response to monosaccharide, nitrogen metabolism, RNA processing and chloroplast. Many GO categories related to flavor development were overrepresented (e.g. fatty acid catabolism, amino acid metabolism, alcohol metabolism and isoprenoid metabolism).

**Fig. 2:**
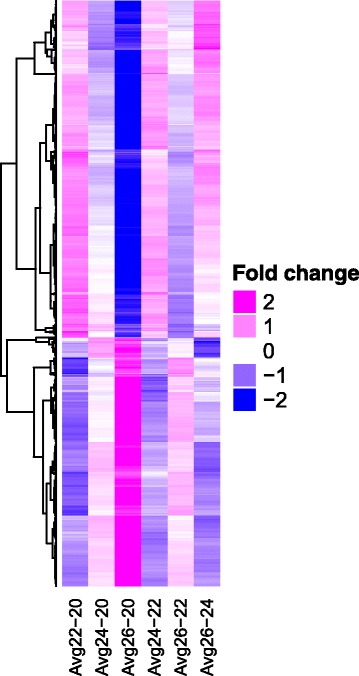
A heat map representing the difference in log2 expression of the 2108 DEGs with different °Brix contrasts. Individual ratios for the six contrasts were computed for each cultivar and then averaged

Of the 2108 DEGs, approximately half of the transcripts increased and the other half decreased with increasing °Brix level (Fig. 2). Gene set enrichment was performed for the gene ontologies of the top 500 DEGs that increased and decreased the most (the difference in transcript abundance between 20° and 26°Brix) to determine the biological processes involved. The top three enriched GO categories (from a total of 105) for the increasing DEGs were nucleic acid metabolism, MCM (minichromosome maintenance) complex and chromosome organization (Additional file 7). Additional interesting categories that were highly enriched were DNA methylation and lipid catabolism. The top three GO categories (from a total of 197) for the decreasing DEGs were membrane, cell wall and photosystem (Additional file 8). Other interesting categories included regulation of hormone levels and pigment accumulation.

**Fig. 3:**
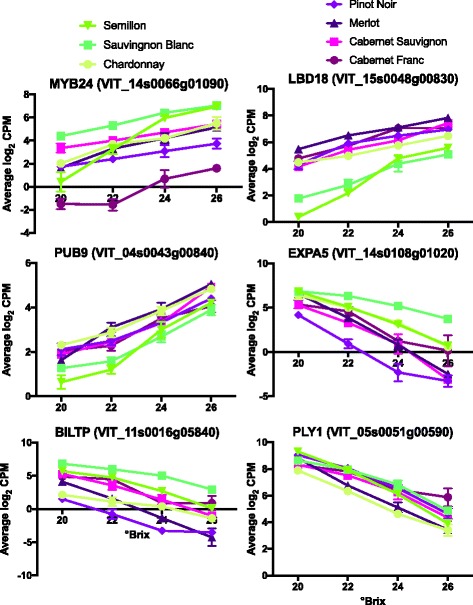
The transcript abundance of some of the top DEGs. Data shown are the means ± SE; *n* = 3. *Error bars* not shown are smaller than the symbol. The symbol legend is displayed in the figure

Examples of three of the top genes with increased and decreased transcript abundance with increasing °Brix level include a MYB transcription factor, a LOB domain protein, a ubiquitinase, an expansin, a bifunctional lipid transfer protein and a pectin lyase (Fig. 3).

### Chromosome organization and regulation of transcription

**Fig. 4:**
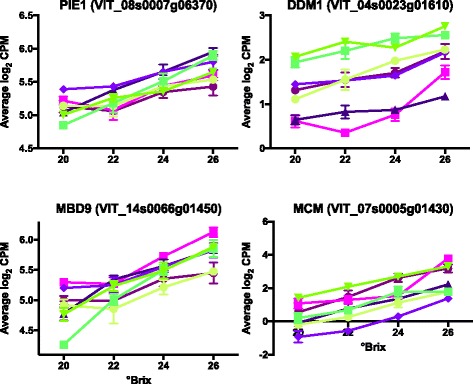
Expression profiles of some genes involved in chromosome organization. Data shown are the means ± SE; *n* = 3. *Error bars* not shown are smaller than the symbol. Symbol legend is the same as in Fig. 3

Gene ontology enrichment analysis indicated that chromosome related events (chromosome organization, DNA and histone methylation, etc.) were significantly modified in the late stages of ripening (Additional file 5). Chromatin remodeling was recently discovered to be very important in regulating tomato fruit ripening [17]. Many genes related to chromatin silencing or chromosome organization that negatively regulate gene transcription were associated with increasing °Brix. These included histone methyltransferases and a number of sucrose non-fermentable 2 (SNF2) transcripts. SNF2 domain-containing proteins participate in epigenetic regulation of gene transcription to control development in plants and other organisms [35]. For example, the transcript abundance of Photoperiod Independent Early Flowering 1 (*PIE1*; VIT_08s0007g06370) whose protein contains helicase and SNF2 domains, was increased with °Brix level (Fig. 4 **)**. Similarly, the transcript abundance of the transcription factor *VviDDM1* (Decrease in DNA Methylation 1; VIT_04s0023g01610) also peaked at 26 °Brix. *DDM1* belongs to the Lsh subfamily of SNF2 domain proteins [35, 36]. The transcript abundance of another TF, methyl-CPG-binding Domain 9-like (*MBD9;* VIT_14s0066g01450) also significantly increased with °Brix. And finally, a minichromosome maintenance family protein (*MCM*; VIT_07s0005g01430) increased with increasing °Brix.

### Regulation of hormone level genes

**Fig. 5:**
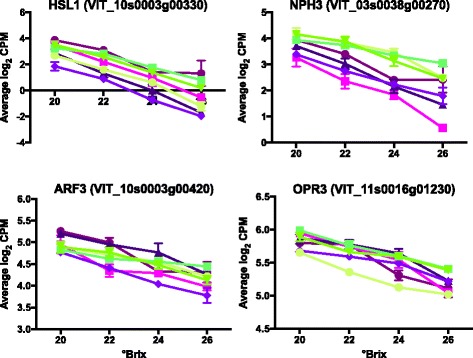
The transcript abundance of some top genes that are involved in the regulation of hormones. Data shown are the means ± SE; *n* = 3. *Error bars* not shown are smaller than the symbol. Symbol legend is displayed in Fig. 3

Genes involved in the regulation of hormones were also significantly enriched. The regulation of several different hormone pathways was represented in this group (Fig. 5). A protein kinase receptor, *HSL1* (HAESA-like 1; VIT_10s0003g00330) is one of the most significantly reduced DEGs with increasing °Brix. It interacts with an abscission signaling peptide to inhibit seed maturation [37, 38]. Its expression is dependent on sugar levels and it also interacts with sugar-inducible and ABA-regulated genes [39, 40]. A reduction in transcript abundance of *VviHSL1* may contribute to seed maturation.

Other genes with decreasing transcript abundance (Fig. 5) included *Non-Phototropic Hypocotyl 3* (*NPH3*; VIT_03s0038g00270), *Auxin Response Factor 3* (*ARF3*; VIT_10s0003g00420), and *Oxophytodienoate-Reductase 3* (*OPR3*; VIT_11s0016g01230). *NPH3* regulates auxin efflux carriers [41] and *OPR3* is involved in jasmonate biosynthesis [42].

### Light responsive and photosynthetic genes

Many blue-light responsive genes, including *Zeitlupe* (*VviZTL*; VIT_11s0052g00730) and *XAP5 Circadian Timekeeper* (*VviXCT*; VIT_03s0038g01810) had an increasing response to °Brix level. These genes are known to measure day length and adjust the circadian clock. The transcript abundance of a *Constans-Like 4* gene (*VviCOL4*, VIT_04s0008g07340) was increased significantly with °Brix. Constans-like genes were first identified in flowering and are important sensors of day length and light-driven redox signaling [43]. *Constans-like 13* (VIT_07s0104g01360) belongs to Group III of CO-like TFs [44, 45]. Almada et al. [44] reported both spatial and temporal expression patterns for the *VviCOL1*, with a reduction of expression in maturing berries, a pattern seen in all cultivars.

Nearly all of the transcripts for photosynthetic DEGs were decreasing with increasing °Brix level, such as *Cytochrome C6A* (VIT_01s0011g01850) or *light harvesting complex II type I CAB-1* (VIT_10s0003g02890). The gene expression of some of these photosystem genes appears to be completely shutting down. Only two transcripts associated with photosynthesis were increasing in expression: a pentatricopeptide repeat-containing (PPR) protein (VIT_03s0063g00900) and ferritin (VIT_08s0058g00440). These results support the hypothesis that chloroplasts are becoming nonfunctional for photosynthesis or possibly even degraded. Chloroplasts are also the location for isoprenoid, carotenoid and terpenoid metabolism, and thus a source of important volatiles and aromas [46].

### Gene co-expression network analyses

#### WGCNA

WGCNA was applied to the entire set of transcripts to systematically and globally identify highly-connected gene subnetworks or module eigengenes (MEs), which are the first right-singular vectors of the standardized module expression data. WGCNA was also used to identify highly correlated hub genes within these modules. Results from the WGCNA R package hierarchical clustering function confirmed the PCA results that the transcript abundance of all quality-controlled transcripts of the berry samples separated very well according to cultivar and °Brix effects (Additional file 9). WGCNA produces module memberships with highly enriched GO categories with important biological meaning [26, 32]. WGCNA defines modules as clusters of gene nodes with high topological overlap, which means that members of a given module share a greater number of connections with other members of the module than with genes outside the module [27]. The gene network had near scale-free topology (Additional file 10) with a number of highly connected hub gene nodes. Extensive branches (clusters of transcripts forming modules) can be observed in the gene dendrogram produced using average linkage hierarchical clustering (Additional file 9). Utilizing the WGCNA R package, 64 modules were identified by applying the cuttreeDynamic function with a minimum membership of 30 genes. Modules were merged based on a threshold of 0.25 (see Methods for details and the colored blocks in the merged dynamic in Additional file 9). The color grey is reserved for genes not belonging to any module, thus the grey module (MEgrey) does not present a real module.

**Fig. 6:**
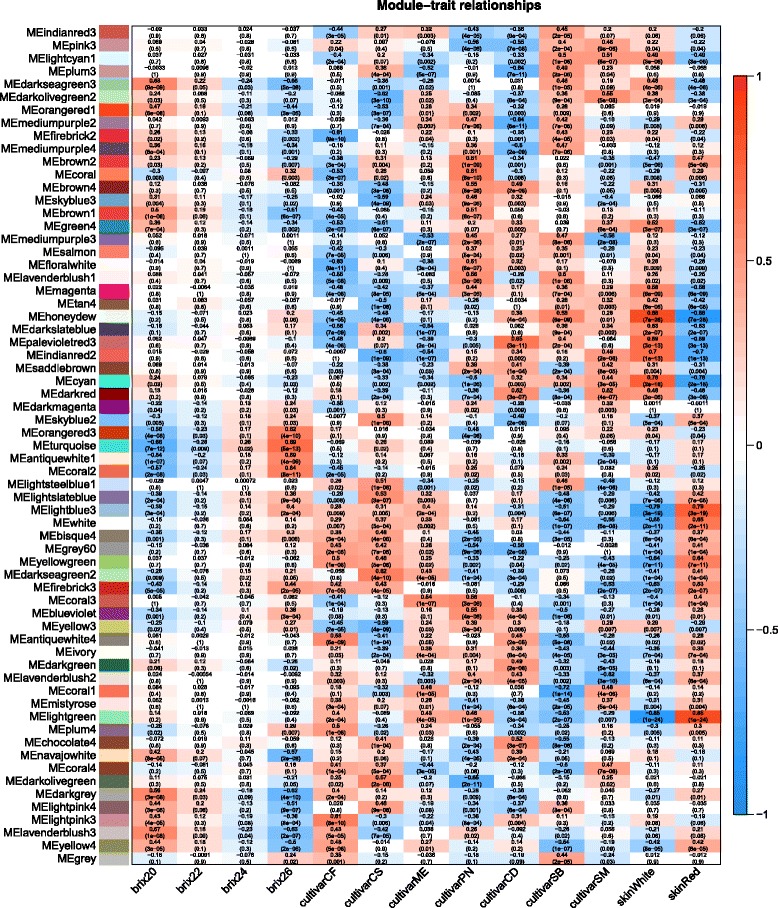
Heatmap correlation of berry traits (°Brix level, cultivar, red vs white grape) of each of 64 gene modules. Gene modules were identified by a color name (MEcolornumber) as assigned by the WGCNA R package. Values in each heatmap *block* are the correlation (*upper*) and *p*-value (*lower*) of the module eigengene with the berry trait

A module eigengene was calculated for each module and correlated with the berry traits: °Brix level, cultivar, red or white grapes (Fig. 6). Each square in the figure table was colored if it was statistically significant and labeled with two numbers; the upper number is the correlation coefficient and the lower number is the *p*-value. Some modules were significantly correlated with specific traits. Here we focus on modules most correlated with the °Brix levels. The orangered3, turquoise, antiquewhite1 and coral2 were the most significantly correlated modules with an increasing gene expression trend with increasing °Brix (Fig. 6). The darkgrey, navahowhite, darkseagreen3 and lavenderblush3 modules were the most significantly correlated modules with a decreasing gene expression trend with increasing °Brix.

Module membership (kME or the module eigengene connectivity) was calculated for each gene for each module (Additional file 11). A kME value of 1 indicates perfect correlation with the module eigengene and the higher the kME of a gene the higher its connectivity within the module. Genes with a high kME are considered hub genes. GO category enrichment was determined with the top 500 genes of each module (data not presented).

Major details of all 64 modules were summarized (Additional file 12), including the top hub gene and GO categories highly enriched in each module. Half of the 64 modules were significantly correlated with °Brix (Additional file 12). Many of these modules were enriched with many GO categories including chloroplast, ribosome, cytoplasm, nucleus, photosynthesis, translation, nucleic acid metabolism, phenylpropanoid biosynthesis, defense responses, etc.

**Fig. 7:**
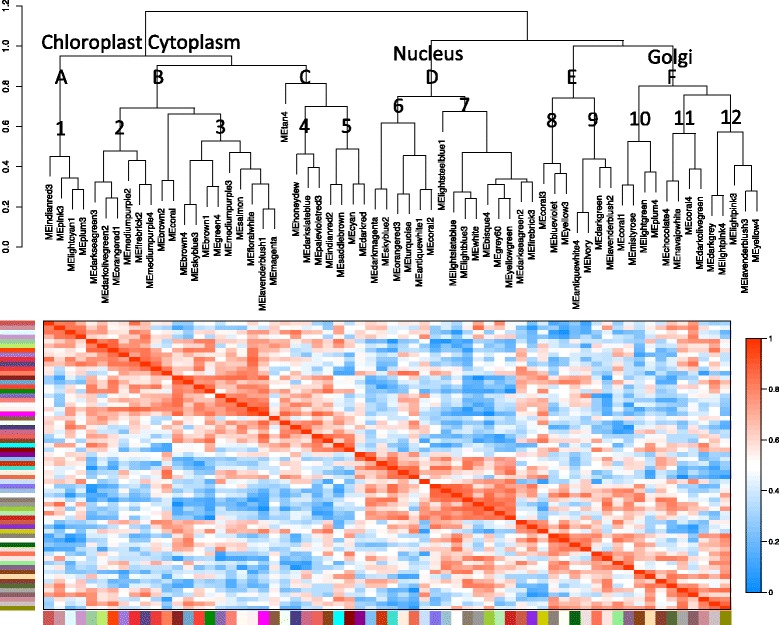
A hierarchical clustering dendrogram and heatmap of module eigengene correlations. The hierarchical clustering dendrogram is overlaid with symbols identifying gene subnetworks. *Colored blocks* at the periphery represent individual modules

Module eigengenes for all 64 modules were correlated with each other to elucidate the relationships between modules (Fig. 7). Hierarchical clustering revealed a complex network order; 6 higher order subnetworks (A to F) could be subdivided into what we call “module subnetworks” (1 to 12). The 12 module subnetworks were identified with a minimum set of three modules (Additional file 12, Fig. 7).

The higher order subnetworks, A, B, and C, all were highly enriched in chloroplast, cytoplasm, photosynthesis and translation GO categories (Additional files 11 and 12). They were mostly associated with higher expression in white grape varieties, but there were a significant number of modules that had no differences between red and white grapes.

Higher order subnetwork D was enriched in the nucleus, nucleotide binding and gene expression. Higher order subnetwork E was not enriched in any particular cellular compartment but was enriched in phenylpropanoid and aromatic compound metabolism GO categories. Higher order subnetwork F was enriched in the golgi apparatus and transport processes. These latter higher order subnetworks (D-F) were mostly associated with higher expression in red grape varieties, but there were a significant number of modules that had no differences between red and white grapes (Additional file 12); these represent common gene subnetworks for all cultivars.

Some module subnetworks were large and complex, consisting of 8 or 9 modules (Additional file 12, Fig. 7); they formed a subnetwork within a larger subnetwork. For example, the Group 3 module subnetwork consisted of modules involved in protein folding, translation, chloroplast and response to light. Only 3 of the 8 modules in this subnetwork were correlated (negatively) with increasing °Brix, the rest were more correlated with different genotypes (Additional file 12, Fig. 6).

The Group 2 module subnetwork consisted of 6 modules and was similar to Group 3; the module clustering in the module dendrogram indicated that the subnetworks formed into a larger branch or subnetwork. Five of the six modules in Group 2 negatively correlated with increasing °Brix. These modules had gene ontologies involving the chloroplast, translation, and photosynthesis and were similar to those in Group 3.

In summary, the WGCNA network of the grape berry skin at the late ripening stages could be divided into 6 higher order subnetworks and 12 module subnetworks. These subnetworks were enriched in GO categories involving cellular compartmentation (e.g. chloroplast and nucleus) and major metabolic processes (e.g. photosynthesis, translation, and phenylpropanoid metabolism). In the next section we will focus on details in the two largest gene modules identified by WGCNA that were correlated with °Brix levels.

### Turquoise module had the largest positive correlation with Brix

The turquoise module was highly correlated with three other modules: orangered3, antiquewhite1 and coral2 (Fig. 7). These four modules formed the core of the Group 6 module subnetwork (Fig. 7). There were many common GO categories amongst these modules including nucleic acid and protein binding (Additional file 12). Interspersed within the top kMEs were many of the core circadian clock genes (Additional file 11).

The turquoise module eigengene had the highest significant correlation of 0.69 (*p*-value = 4e-10; Additional file 12; Fig. 6) with 26 °Brix. The turquoise module was the largest most connected module with 355 transcripts having a 0.80 kME or higher. Transcript abundance in this module increased with °Brix level. The response was generally common for all varieties (*p*-value >0.1 for red vs white grape and all varieties except Cabernet Sauvignon). The top hub was VIT_13s0067g03760, a pre-mRNA-splicing factor 3 protein, with a kME of 0.96. The protein of this gene is required for RNA-directed methylation of DNA [47].

There were 337 GO categories overrepresented in the top 500 genes in this module (data not shown). Highly enriched GO categories of the top 500 turquoise module genes included nucleic acid binding (*p*-value = 1.74e-17), nucleus (2.86e-15), mRNA metabolic process (9.28e-17), and protein binding (1.11e-11). Other interesting GO categories included RNA processing, epigenetic regulation of gene expression, post-embryogenic development, response to red or far red light and autophagy.

The top 100 genes of this module were highly enriched in specific categories that may be interacting with each other; 32 were involved in RNA processing, 22 were involved in chromosome organization, 12 were involved in proteolysis, 9 were transcription factors, 2 were involved in the circadian clock or circadian rhythm and 2 were involved in autophagy (See Additional file 13 for color highlights and a list of genes in each category). Many of these processes are intimately connected to the core circadian clock (see Discussion). These genes represented 79 of the total top 100 hubs, indicating very high enrichment of these GO categories in the top 100 genes.

Some of these top 100 kME genes were involved in more than one category. For example, CBF1-interacting co-repressor CIR domain containing protein (VIT_02s0087g00830; hub rank #43), Enhancer of polycomb-like transcription factor protein (VIT_04s0008g04370; hub #67), *Microrchidia 4* (*MORC4*; VIT_17s0000g00910; hub #75), *RNA Polymerase II large subunit* (VIT_18s0001g00860; hub #89), *High Mobility Group* (*HMG*; VIT_14s0108g00040; hub #92) are involved in both RNA processing and epigenetic regulation; *methyl-CPG-binding domain 9* (*MBD9*; VIT_14s0066g01450; hub #25) and *DDB1-CUL4 associated factor 1* (VIT_04s0008g03060, hub #9) are involved in RNA processing and protein ubiquitination.

The turquoise module was highly connected to the circadian clock and other light regulated genes; the module contained the largest number of circadian clock and light-regulated genes (16) in the top 500 kMEs (positively correlated) than any other module (Additional file 11); it also had 5 circadian clock and light-regulated genes in the bottom 500 kMEs (negatively correlated), inferring that these genes may have been negatively regulated or repressed by the same factors that positively regulated the genes within the turquoise module. The light-regulated gene from the turquoise module with the highest kME was *COP1-interacting protein 4.1* (*CIP4*; VIT_01s0137g00190; 0.91 kME, hub #37). *CIP4* was highly connected in 3 similar modules: its hub rank was equal to #37, #214, and #282 for turquoise, orangered3, and coral2 modules, respectively. *CIP4* is a transcriptional activator that promotes photomorphogenesis [48]. It appears to act downstream of most photoreceptors and Constitutive Photomorphogenic 1 (*COP1*). Photoreceptors can inhibit *COP1* and thus activate *CIP4*.

The next highest hub in this group was Time for Coffee (*TIC*; VIT_05s0020g03150; 0.89 kME; hub #55); it is the circadian clock gene with the highest connectivity. It interacts with *LHY* (Late Elongated Hypocotyl) and *PRR9* (Pseudo-Response Regulator 9) in the core circadian clock [49]. *ZTL*, also known as Adagio Protein 1 (VIT_11s0052g00730), was the next most connected circadian clock hub gene, having a kME of 0.87 and had a rank of 101 in the module.

*ELF3*, is part of the evening complex (*ELF3*, *Early Flowering 4* (*ELF4)* and Phytoclock 1 (*PCL1*)) and appears to be a key regulatory hub for the circadian clock that modulates light signals [49]. The gene expression trends for *Far-Red Impaired Response 1* (*FAR1*) and other FAR1-related genes, *ELF3*, *ZTL*, *Topless* (*TPL)*, and *COP1* within our data set were very similar and indicate a possible light signal sensing cascade/cycle involving *FAR1* (or a FAR1- related sequence protein), *PHYB* (phytochrome B), *ZTL*, *ELF3, COP1 and TPL*.

*TPL* was another interesting gene from the circadian clock core in this subnetwork of increasing gene expression; it forms a complex with *PRR9* and *HDA6*, a histone deacetylase [50] that positively regulates chromosome compaction. *TPL* is a broad repressor of many genes and down-regulates the early morning genes as a co-repressor with *PRR9*, which binds to the promoters of the morning genes, *CCA1* (Circadian Clock Associated 1) and *LHY*, inhibiting their expression. Increasing *TPL* shortens the day phase (and lengthens the night phase) as in short days. It also interacts directly with *EMF1* (Embryonic Flower 1) and *WRKY32* [51]; both of these genes were within the top 100 kMEs of this module. *EMF1* (VIT_04s0008g03660; 0.88 kME; hub #74) is part of a Polycomb group (PcG) complex (chromatin modification mediating transcriptional repression) and regulates flowering by repressing *Flowering Locus T* (*FT*) expression; it helps to synchronize environmental cues and is a vascular signal.

Another top hub, Early Flowering in Short Days (*EFS*; VIT_18s0001g01700; 0.93, 17) is a histone lysine N-methyltransferase required specifically for the trimethylation of H3-K4 in *Flowering Locus C* (*FLC*) chromatin. It also affects carotenoid biosynthesis genes, and light and carbon responsive genes. *EFS* methylates *LHY* in Arabidopsis [52]. Other top hub genes were histone ubiquitin ligases (HUBs) involved in protein processing and post-embryonic development. Monoubiquination of histones by HUBs stimulates gene expression [53]. Thus, there is a strong representation of key components of the circadian clock in this gene module.

There were 4 autophagy genes in the top 500 kMEs of the turquoise module: homolog of yeast autophagy 18 G (*ATG18g*), autophagy 9 (*APG9*), autophagy-related 11 (*ATG11*) and autophagy 2 (*ATG2*). In particular, 3 of the 4 are part of the *APG9* cycling. *ATG18g* (hub #20) was the most highly connected autophagy hub followed by *APG9* (hub # 122). Autophagy is an important part of the senescence process [54, 55].

### Darkseagreen3 module had the largest negative correlation with °Brix

The darkseagreen3 module was part of the Group 2 module subnetwork and thus a part of one of the largest subnetworks elucidated in the late stages of berry ripening. The darkseagreen3 module eigengene had a significant correlation of −0.55 (*p*-value = 5e-08) with 26 °Brix. It was the second largest most connected module with 342 transcripts having a 0.80 kME or higher. The transcript abundance of gene members in this module decreased with increasing °Brix level. This decreasing response was generally common for all varieties, but there was higher transcript abundance in white grape skins. The top hub was VIT_09s0002g04360, a CURvature Thylakoid1 protein (*CURT1*), with a kME of 0.96, a membrane phosphoprotein responsible for curvature at the grana margins [56].

There were 422 GO categories overrepresented. The top GO categories of the top 500 kMEs were chloroplast (2.95e-47), thylakoid (6.29e-46), photosynthesis (3.59e-32), and cytoplasm (4.27e-29). Other interesting GO categories included isopentenyl diphosphate biosynthetic process, mevalonate-independent pathway, alcohol metabolic process, monosaccharide metabolic process, lipid biosynthesis, and cysteine biosynthesis.

There were 7 and 8 circadian-related and light regulated genes in the top and bottom 500 kMEs, respectively. *ELF4*, part of the evening complex, was the top circadian clock gene at #106, another circadian rhythm gene was *Plastid Transcriptionally Active 16* (*PTAC16*; VIT_06s0004g05230; 0.86 kME) at #113, *Transcription factor TCP domain protein 7* (*TCP7*; VIT_10s0042g00170; hub #180), *Accumulation and Replication of Chloroplast 5* (*ARC5*; VIT_12s0055g00490; hub #221), *Light-Inducible And Clock-Regulated 2* (*LNK2*; VIT_13s0139g00360; hub #223), and *Regulator of Chromosome Condensation* (*RCC1*; VIT_07s0031g02560; hub #455).

Some of the top negatively correlated circadian clock genes included *VviELF3*, *VviZTL*, *VviSKIP* (SNW/SKI-Interacting Protein; VIT_11s0016g03290), *VviTIC* (VIT_05s0020g03150), *VviRVE1* (ReVeillE 1; VIT_04s0079g00410) and *VviFRS5* (FAR1-Related Sequence 5; VIT_03s0038g04140). These genes were all positively correlated hubs in the turquoise module and indicated a coordinated regulation between the turquoise and darkseagreen3 module.

### Petal *analysis*

To provide another independent approach for gene co-expression analysis, petal analysis was performed on the same set of 16,606 filtered and quality-controlled genes. Petal constructs models based on seven different Spearman correlation thresholds and calculates multiple topological network measures to automatically determine the ‘best’ possible network model for this dataset meeting scale-free and small-world characteristics [29]. The final model used for down-stream analysis (module and hub identification) is constructed based on a Spearman correlation value of 0.703 and includes 15,092 of the 16,606 original genes (Additional file 14). Here we define hub genes by the top 5% of connected genes within the final network model corresponding to 751 genes; their connectivity (k) ranged between 395 and 788. All maximal largest cliques within the hub subnetwork were extracted, resulting in 90 cliques (completely connected subnetworks) with 89 genes each. The intersection and union of these largest cliques include 74 and 105 genes, respectively. The union is a highly intra-connected subnetwork of 105 genes with a density of 0.99 (i.e., 46 edges (links) are missing to make a clique).

To build a larger subnetwork, the common immediate neighbors of the 105 hub genes were combined into what we define as a hub module; this resulted in a module of 216 genes with density of 0.85 (Additional file 15). The genes in this hub module (216 genes, Additional file 15) were also represented in the WGCNA turquoise module. Both approaches independently identified gene modules with highly connected hub nodes, although WGCNA’s turquoise module included more genes, its intra-connectivity was much lower, indicating that the gene expression profiles within the WGCNA turquoise module exhibited greater variability compared to petal’s hub module. There was general agreement of the order of hubs in petal with the kME values of the hubs in the turquoise module (Additional file 15). A true comparison is difficult, because WGCNA’s kME values are local hub measures, whereas petal defines hubs based upon the entire small-world and scale-free network model. Thus, we can conclude that the two gene co-expression network approaches consistently identified a list of highly connected hubs in a common network of all grape cultivars of the late stages of grape berry ripening.

### Integration of WGCNA module subnetworks with the circadian clock in berry skins

**Fig. 8:**
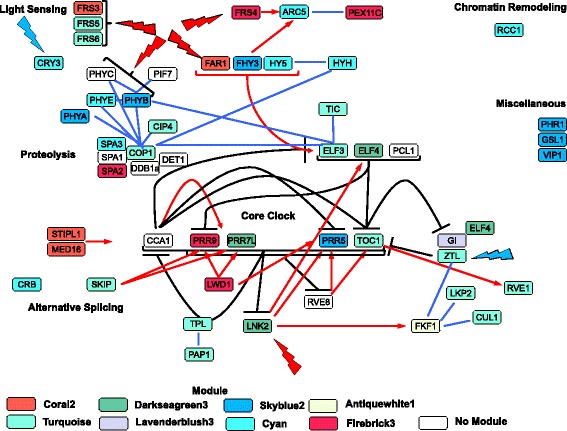
An integrated circadian clock model with gene modules of grape berry skins in the late ripening stages. *Colored symbols* indicate genes within that particular module. Light sensing, proteolysis, alternative splicing and chromatin remodeling or miscellaneous represent certain regions of the model. *Red* and *blue* lightning bolts represent the reception of their respective light wavelengths for each gene symbol. *Lines* in the model represent known interactions between genes; *red arrows* are *positive* interactions, *black lines* are *negative* interactions, and *blue lines* indicate *direct* physical interactions but the direction, *positive* or *negative*, is unknown. *No lines* indicate that there are no known interactions at this time

Several other WGCNA modules had circadian clock genes in the top (most positive) or lower (most negative) 500 kMEs. To better understand the role of the circadian clock in fruit ripening, an integrated circadian clock model was constructed relating known circadian clock genes with the grape berry skin gene modules in which they had the highest correlation (Fig. 8). The model integrated both blue and red light receptors (identified with red and blue lightning bolts) as well as genes involved in biochemical processes regulating the clock. These processes included light sensing, proteolysis, alternative splicing and chromatin remodeling. The model depicted a complex interplay of these processes and the large number of gene modules that were affected. Lines in the model represent known interactions between genes; red arrows are positive interactions, black lines are negative interactions, and blue lines indicate direct physical interactions but the direction, positive or negative, is unknown. No lines indicate that there are no known interactions at this time.

From this model it is hypothesized that light signals (in this case a shortening of the day length with a daily sunrise occurring later each day as time passes in Autumn and °Brix increases in the berry) drive the resetting of the clock through the photoreceptors. These in turn affect a series of positive and negative feedback loops in the model.

More subtle associations in the model can be perceived through the module correlations. The two modules participating the most in the clock were the turquoise (14 members) and cyan (8 members) modules. *ZTL*, *FRS5* and *FRS6* photoreceptors were part of the turquoise module. The Cryptochrome 3 (*CRY3)* photoreceptor was part of the cyan module. There is no direct interaction evidence of these photoreceptors to the core clock components, but their expression was highly correlated with some clock components (e.g. *HY5 (Elongated Hypocotyl 5)*, *ELF3*, *COP1*, *PHYE (Phytochrome E)*, *SPA3 (suppressor of phytochrome A-related 3*, etc.) indicating some sort of linkage. In other cases, module associations were more obvious. For example, *ZTL*, a blue light photoreceptor, has a number of known interactions with clock components, including the inhibition of *TOC1*. *LNK2* has direct positive effects on *ELF4*.

At a higher gene subnetwork level, module subnetwork 6 was largely represented by circadian clock components in the turquoise, coral2, antiquewhite1 and skyblue2 modules. Again, these modules were enriched in the nucleus, RNA splicing, chromosome organization, epigenetic regulation, and ubiquitination. The circadian clock components of the darkseagreen3, cyan, firebrick3 and lavenderblush3 modules were each part of the module subnetworks 2, 5, 7 and 12, respectively. These subnetworks were enriched in the chloroplast and the cytoplasm categories.

Thus, this model depicted different genes (or proteins) within the circadian clock that had direct positive and negative interactions with other genes or proteins in these subnetworks, reflecting the complex interplay of positive and negative feedback loops in the circadian clock. Other genes were connected in this clock model through gene module associations, but with no known physical interactions. These genes are new targets for future research on the nature of their interactions within the clock.

## Discussion

### The mature berry skin transcriptome was highly dynamic

This study investigated grape berry skins sampled at four concentrations of total soluble sugars: 20, 22, 24 and 26 °Brix. All of these grapes were grown in the same vineyard and thus exposed to nearly identical environmental conditions. We sampled individual berries from a cluster over a narrow developmental range of increasing sugar in an attempt to reduce variability of our samples. Most of the transcriptional changes could be examined with co-expression subnetworks and could be associated with highly enriched GO categories. By comparing both red and white grape cultivars in the same vineyard we could identify modules common to all cultivars that were active in the late ripening stages. In the following sections we discuss some of the processes that appear to be involved in the berry skin gene subnetworks during the late stages of ripening.

### Accumulation of sugar and gene expression

Sugar levels appear to play a role in the transcriptional profiles of the berry skin. PCA and the hclust function utilized in the WGCNA R package showed clear separation by sugar level and a uniformity between samples. These observations were similar to other studies where developmental stages were separated by °Brix [57–60]. Grape berries on a cluster can ripen asynchronously with a range of °Brix levels [61]. The lack of uniformity in sugar concentration can range from 5 to 7 °Brix within a grape cluster [62]. Indeed, we observed varying °Brix within a cluster while separating individual berries from our clusters in this study, which followed a normal distribution within a cluster. Ripening related asynchronicity within a cluster has been shown to synchronize in some situations at maturity [60], but this process was not complete in our berries of seven different varieties, where we observed differences in berries on a single cluster of approximately 4 °Brix (sometimes a difference of 8 °Brix was observed).

Gene expression in grape berries is affected by the sugar concentration [16]. Grape berry sugar concentrations increase substantially after veraison, when soluble sugars are actively transported via the phloem while vines are photosynthetically active [7, 63, 64]. Sugars influence fruit development and gene expression in other plant species [11–13]. In grapes, sugar can affect cell growth and induce the transcription of some genes in berries [16]. Sugar increases the expression of a glucose-6-phosphate transporter facilitating sucrose transport for starch conversion in plastids [65] and acts as a sensor for sugar signaling [10]. A putative glucose-6-phosphate transporter (VIT_19s0177g00300) had increasing transcript abundance with °Brix (Additional file 3).

Some bZIP TFs also contain a sucrose-controlled upstream open-reading frame that exhibits repressed expression under increasing molarities of sugar [66, 67]. The promoter sequence of a dihydroflavonol reductase gene (VIT_18s0001g12800) contains a G-box binding domain, MYB and sucrose box domains that can be induced by sucrose, glucose and fructose, constituents of a ripening berry [68]. Sugar regulates other genes involved in sugar transport and anthocyanin biosynthesis in grape berries [16].

Thus we confirmed our hypothesis that the transcript abundance of genes would vary with sugar level. This argument is supported by the substantial separation of samples by sugar level in the principal component 2 (Fig. 1), which was only slightly lower (21.4%) than principal component 1 (21.8%), which separated by genotype. In addition, there was an enrichment of several GO categories for responses to sugar (Additional files 5 and 6). While harvesting grapes at different times may have contributed to some variability in the samples, separating samples based on their sugar level captured a large part of the variability. It is likely that some changes in gene expression in these berry skins were influenced by the sugar concentration.

### Autophagy and the decrease of photosynthesis transcripts

Many ripening related processes were observed in our data. There was an increase in specific hub genes involved in autophagy. The most highly connected hub for autophagy was the *ATG18g* transcript in the turquoise module (hub #20; Additional file 11). Autophagy is a degradative process that involves the formation of autophagosomes [54] and intracellular vesicle transport [55]. *ATG18g*, *APG9* (*ATG9*) and *ATG2*, all hubs in the turquoise module, are part of the *ATG9* cycling system, which participates in the formation of autophagosomes [54]. There were also a number of proteolysis genes in the top 100 hubs of the turquoise module (Additional file 13). Other senescent-like processes have been observed during grape ripening such as chloroplast disintegration [23, 24] and cell death [22].

Ripening also included the continued decrease in transcript abundance of most photosynthetic transcripts that were highly enriched in the darkseagreen3 module. This is consistent with the deactivation or degradation of chloroplasts; interestingly, the darkseagreen3 module was negatively correlated with the turquoise module indicating that there may be an interaction between the two subnetworks.

Most photosynthesis-related transcripts were decreased in late ripening berries. The ripening berry is a sink organ for photosynthate, losing its photosynthetic capacity with time and changing color as chloroplasts are degraded or converted to other plastids with changing carotenoid production [46, 69]. In tomato, chloroplasts are converted to chromoplasts, which are the source of the red pigments. In grapes, it appears from two studies that the chloroplasts remain chloroplasts, but that the chloroplasts structure begins to become disorganized with plastoglobules forming and enlarging [23, 24]. These plastoglobules may be a source for lipids used in volatile production during the late stages of fruit ripening [24]. Hardie et al. [24] associated changes in chloroplast structure with the formation of lipid bodies and monoterpenes, which contribute important volatile aromas in maturing grapes. High sugar concentrations can induce senescence [9], repress transcription of the plastome, reduce chloroplast numbers and alter chloroplast morphology [10]. Perhaps the high sugars in the berries have the same effect?

Other ripening processes included cell wall and lipid metabolism. Some cell wall softening genes like a polygalacturonase (VIT_16s0050g01110) and a xyloglucan endotransglycosylase/hydrolase (VIT_02s0012g02220) were positively correlated with °Brix levels in the mature berry skins, whereas the transcript abundance of other cell wall proteins were decreased, such as a group of expansins (Additional file 3). Genes involved in fatty acid and lipid oxidation were also highly enriched in the turquoise module (Additional file 11); the transcript abundance of these genes increased with increasing sugar level.

### Epigenetic regulation

The turquoise module was highly enriched with genes involved with epigenetic regulation. DNA methylation plays an indispensable role in regulating endogenous gene transcription [70]. In general, methylation of genes inhibits gene expression or methylation of RNA affects alternative splicing. Methylation of histones can be permissive for gene expression (e.g. H3K4me3, H3K36me/me2/me3, and H3K9me3). Acetylation and monoubiquination of histones are also generally associated positively with gene activity. In contrast, histone H2A monoubiquitination [71] and H3K27 trimethylation [72] potently repress transcription by the action of the polycomb repressive complexes, PRC2 and PRC1.

Regulation of fruit ripening is linked to DNA methylation. For example, gradual decreases in methylation of the promoter region of the *RIN* MADS-box TF in tomato [17] allows ripening to proceed. Differentially expressed methyltransferases, like *CMT*, *DRM* and *MET*, are transcriptionally active during fruit development in pear [73] and legume [74]. These findings indicate a possible role for normal fruit ripening through the regulation of DNA methylation, particularly in this class of genes highly conserved in eukaryotic species [75].

Methyl-CpG-Binding Domain 9 (*MBD9*) in *Arabidopsis* can modulate DNA methylation and histone acetylation to regulate both flowering time and shoot branching by specifically binding methylated CpG dinucleotides [76–78]. *Atmbd9* mutants flower earlier and show abnormal axillary bud outgrowth [77], displaying significantly methylated promoter and intronic regions of the *FLC* gene [78]. A common increase in transcript abundance of *VviMBD9* in all grape cultivars was observed and raises the possibility for methylation of DNA and histones (which presumably causes a reduction in transcription).

Likewise, *DDM1* and *PIE1* transcript abundance increased in berries with increasing °Brix level in the late ripening stages, peaking at 26° Brix. *DDM1* proteins have been observed co-localizing with *MBD* proteins forming protein complexes [76]. *DDM1* in *Arabidopsis* [79, 80] and rice [81] is necessary for genomic DNA methylation and chromatin remodeling through preferential methylation of histone H3 lysine 9 (H3-K9) instead of transposable elements.

Genome-wide reduction of DNA methylation results in severe developmental and morphological defects in *ddm1* mutants [82]. In *Arabidopsis*, PIE1 forms part of the Swr1-like complex which deposits a histone variant, H2A.Z, onto chromatin around both the transcriptional start and stop sites on genes responsible for flowering repression (*FLC*, *MAF4 (MADS Affecting Flowering 4*) and *MAF5 (MADS Affecting Flowering 5*)) enabling their competence for activation by other factors [83]. Our results support the hypothesis for a role of epigenetic regulation during the late stages of berry development.

### Light and the core circadian clock

Not all DEGs in the berry appear to be related to the sampled °Brix levels, some genes appear to be influenced by the genotype (Fig. 1). Light may also be having an effect on gene expression. There are at least two publications that provide evidence for a role of light and the circadian clock in grapevine.

In the first, over a thousand genes in two *Vitis vinifera* cultivars were recently observed expressing distinctive circadian rhythms throughout the light-dark cycle [84]. For example, *VviLHY* and *VviTOC1* did not oscillate, whereas *VviRVE1* and *VviELF3* of the core clock genes did display a circadian rhythm in grape [84]. The authors attribute the differences in clock gene expression to grape maintaining a simplified clock in ripening fruit. Furthermore, secondary processes seemed more responsive to circadian oscillation in late ripening stages than primary metabolism, such as the phenolic pathway enzymes: stilbene synthases and phenylalanine ammonia lyase [84, 85].

In the second publication, a genome-wide analysis of the cis-regulatory elements (CREs) of grapevine protein-coding gene promoters was performed [86]. Highly enriched modules or gene networks were identified. There are over 4000 promoters with a circadian clock-associated CRE. The genes with this CRE in their promoters are highly enriched in functional categories associated with abiotic stress, hormone, flavonoid, and isoprenoid metabolism. There are a large number of chalcone synthase, stilbene synthase, and terpene synthase genes in this group. The authors suggested that grapevine might have an “expanded clock regulatory network”. In addition, they linked light- and chloroplast-related genes to the subnetwork of the circadian clock gene, *VviHYH*.

The circadian clock is regulated by light and may have affected gene expression in grape berry skins during the late stages of berry ripening. Day length decreased with advanced berry ripening and the progression of autumn.

Numerous genes in the core circadian clock were highly connected in gene subnetworks in the late ripening stage. Many of the core clock genes displayed similar patterns of expression for all genotypes (Fig. 8). As the day length shortened, it appears that the day phase of the clock accelerated or shortened, allowing a greater involvement of the evening complex and its cyclic repression of the morning genes.

The circadian clock is regulated by a number of negative feedback loops and positive regulators [87, 88]. Its regulation involves a complicated interaction of transcription [89], RNA splicing [90, 91], proteolysis [92] and histone modifications [53, 93]. Light regulates histone modifications, gene expression and higher order chromosome organization [53]. Genes from all of these processes are present in our circadian clock model (Fig. 8).

The circadian clock regulates gene expression throughout the day and night. It regulates the rhythmic variation in photosynthesis, starch accumulation, and starch turnover [94]. A well-supported hypothesis is that the circadian clock regulates starch metabolism in the leaf to maintain sugar metabolism throughout the night. The circadian clock in turn is sensitive to photosynthesis and soluble sugar accumulation [95]. Sugars accelerate the day phase of the circadian clock.

Do sugars accelerate the day phase in berries? The role of the circadian clock is well studied in leaves, roots, apical meristems, buds and flowers, but there are few papers on the role of the circadian clock in fruit development.

Light signals set the circadian clock (Fig. 8) as the day length shortens in autumn. The circadian clock likely received day length signals through far red (e.g. FAR1-related proteins) and blue light sensors (e.g. *ZTL*) that trigger changes in gene expression involving RNA processing, proteolysis, chromosome modification and organization, and may lead to major changes in berry metabolism (e.g. suppression of gene expression of chloroplast genes).

Yet berries develop normally in grapevines grown under constant environmental conditions in the greenhouse [96, 97] or growth chamber [11]. Elevated CO_2_ concentrations substantially accelerate photosynthesis and berry development [97], indicating that sugar accumulation may affect rates of berry development. Can changes in day length or the circadian clock alter the rate of berry development and fruit composition as well? To what extent the circadian clock functions in berry development is unclear, but the network analyses results in this study indicate that it was highly connected to major developmental processes and gene modules related to epigenetic regulation and chromosome organization. The interactions of sugar with the circadian clock may be even more complex. The roles and interactions of the circadian clock with sugar accumulation and grape berry development warrants further investigation.

## Conclusions

Gene expression of berry skins in the late stages of ripening was associated with sugar accumulation and genotype. A common set of genes for all seven cultivars was identified in the late ripening stages of berry development. Transcriptional regulation of fruit ripening involved many transcription factors and other regulators of hormone levels. In addition, the transcript abundance of genes related to DNA methylation indicated that epigenetic programming might be involved in the regulation of transcription during berry ripening at maturity, suppressing or silencing many genes. Gene co-expression analysis was used by two different approaches to elucidate complex transcriptional networks. Two of the most highly connected gene subnetworks consisted of hundreds of genes: one subnetwork involved RNA processing, chromosome organization, epigenetic regulation, proteolysis and autophagy and the other subnetwork, negatively correlated with the first, involved photosynthesis. Thus, there seems to be an interaction between these two subnetworks that links nucleotide metabolism and autophagy with berry skin ripening processes including the large decrease in photosynthetic transcripts. A circadian clock signature for key clock components was also observed to participate in these subnetworks and warrants further study to better understand the role light plays in these subnetworks and the late stages of berry ripening.

The identification of a core set of genes common to all seven cultivars, both red and white wine producing, allowed the identification of key processes in the development of late ripening berry skins, including autophagy, catabolism, nucleotide metabolism, photosynthesis, cell wall metabolism, gene expression and chromosome organization. Some of these key processes were associated with specific modules or gene subnetworks and the key hub genes in these subnetworks were ranked for future targeted testing. These transcriptomic results support the hypothesis that senescent-like processes dominate the late stages of berry ripening as the seed matures and prepares for dispersal.

## Methods

### Plant materials

*Vitis vinifera* L. cultivars Cabernet Franc, Cabernet Sauvignon, Merlot, Pinot Noir, Chardonnay, Sauvignon Blanc and Semillon were grown at the University of Nevada, Reno’s Nevada Agricultural Experiment Station Valley Road Experimental Vineyard. Research approval was obtained by Grant R. Cramer from the Nevada Agricultural Experiment Station and the University of Nevada, Reno. All grape cultivars were originally obtained as certified material from Inland Desert Nursery, Benton, City, Washington, USA. The cultivars were surveyed in September and October 2012, depending upon the berry maturity of each cultivar. Maturity was assessed using a digital refractometer (HI 96811, Hanna Instruments, Woonsocket, RI, USA) to measure soluble solids (°Brix) that are mostly made up of sugars. Berry clusters were collected between 11.00 h and 13.00 h (near solar noon) in an attempt to minimize temporal transcriptional response variations caused by the circadian clock. At harvest, individual berry °Brix levels were determined with a digital refractometer. Separated berry skins were placed into 50 mL centrifuge tubes in liquid nitrogen according to sugar level (1 ± 0.5 °Brix increments; 19 to 27 °Brix). Berry to berry variation was considerable on an individual cluster; in some cases berries varied as much as 8 °Brix on a single cluster. In this way berries were collected over many days from multiple clusters from multiple vines from 3 different individually irrigated blocks in the vineyard. Each block was considered an experimental replicate.

### RNA extraction

Three experimental replicates from each cultivar at 20, 22, 24 and 26 °Brix were used for sequencing. Total RNA was extracted from approximately 250 mg of finely ground skin tissue using a modified CTAB extraction protocol followed by an additional on-column DNase digestion using a Qiagen RNeasy Mini Kit (Qiagen, Valencia, CA, USA) as in [33]. RNA quality and quantity were assessed with a Nanodrop ND-1000 spectrophotometer (ThermoFisher Scientific, Waltham, MA, USA) and an Agilent 2100 Bioanalyzer (Agilent Technologies, Santa Clara, CA, USA).

### RNA-seq library preparation and sequencing

Eighty-four 50 bp single-end, barcoded libraries were constructed and sequenced by the Neuroscience Genomics Core at the University of California, Los Angeles (Los Angeles, CA, USA) using Illumina TruSeq RNA library prep kits (Illumina Inc., San Diego, CA, USA) according to the manufacturer’s instructions. The barcoded libraries were pooled and multiplexed, and were sequenced using Illumina TruSeq chemistry (version 3.0) on a HiSeq2000 sequencer (Illumina Inc., San Diego, CA, USA).

### Gene expression and statistical analysis

The single-end sequence fragments (reads) generated by Illumina sequencing were base-called, demultiplexed, and then quality filtered with the NGS QC Toolkit [98]. TopHat2 (version 2.0.10) was used to align reads to the V1 version of the PN40024 *Vitis vinifera* reference genome annotation obtained at EnsemblPlants [99]. Filtered reads were aligned with the “--b2-very-sensitive” option for Tophat2 and “--transcriptome-index” option with a corresponding EnsemblPlants-sourced index, as in [33, 100]. Remaining parameters were kept at default. Approximately 93% of reads were mapped (Additional file 1). Samtools [101] and HTSeq [102] were used to generate feature counts from the BAM alignment files produced by Tophat2. HTSeq was run using the “union” mode, with the “-i gene_id -t exon -s no” options. Counts of genes were filtered prior to expression analysis, using the following independent criteria: transcripts with zero counts in all samples were excluded; further filtering was performed to remove transcripts with <15 counts in 75% of samples (63 of 84) prior to expression comparison (Additional file 2). Correction scaling factors were computed from the filtered and untransformed library counts using the trimmed mean of M-values method between each pair of sample libraries, as implemented in edgeR (3.14.0) [103]. This produced an effective library size for model-based differential expression analysis in edgeR. A design model that defined each °Brix level combination as an element of a group (~ 0 + Group) was used to test for differential expression using simple contrasts between subgroups of interest within each cultivar (e.g. 22–20, 24–20, 24–22, 26–20, 26–22, 26–24). Statistically significant transcript abundance changes were found below the adjusted *p*-value of 0.05, according to the Benjamini and Hochberg procedure to control the false discovery rate (FDR) [104]. An intersection of statistically significant transcripts at different °Brix levels was made to derive a common set of berry skin transcripts shared in the seven grapevine cultivars. RNA-seq statistics are presented in Table 1.

**Table 1:** RNA-sequencing, read mapping and feature count statistics of the experiment

Next-generation sequencing platform	Illumina Hiseq2000
Cultivars investigated	7
Library type	Single-end
Number of libraries	84
Read length (bp)	50
Total number of reads	2,901,803,214
Average total reads	34,545,276.36
Total number of bases (Gb)^a^	145.09
Total number of HQ^b^ filtered reads	2,877,839,522
Average HQ reads	34,259,994.31
Total number of HQ bases in HQ reads (Gb)	143.89
Percentage of HQ filtered reads	99.18%
alignment_not_unique^c^	3,410,081.85
ambiguous^c^	219,495.24
no_feature^c^	2,692,823.24
not_aligned^c^	0.00
too_low_aQual^c^	0.00

^a^Giga base pair (1,000,000,000 bp)

^b^High quality (Phred score = 20)

^c^HTSeq

### Gene co-expression analysis

Gene expression was also evaluated with the WGCNA R package version 1.51 [27] to identify modules of correlated genes and investigate intramodular hub genes. A variance-stabilizing transformation of the filtered counts of genes was performed using DESeq2 version 1.12.3 [105] before WGCNA, as suggested by WGCNA package authors. To meet the scale-free topology criteria for optimal clustering, a power of 11 was selected as a soft threshold value to transform the adjacency matrix (see Additional file 10); a biweight midcorrelation was used as an alternative to Pearson’s correlation. The following settings for the adjacency function (datExpr, power = 11, type = “signed hybrid”, corFnc = “bicor”, corOptions = “use = ‘p’, maxPOutliers = 0.1”) and for the cuttreeDynamic function (dendro = geneTree, distM = dissTOM, deepSplit = 2, pamRespectsDendro = FALSE, minClusterSize = 30); these functions have been shown to be the best approach for biologically meaningful results [26].

The petal R package [29] was independently utilized to construct a true small-world and scale-free complex gene co-expression network model from which gene modules with higher intra-correlation and density were identified. Before creating a network model, the distribution of the entire dataset of 16,606 filtered and quality-controlled transcripts was investigated for normality to ensure statistically appropriate association measures to define genes as similar. The graph generated by graphHistQQFromFile clearly showed a non-normal distribution (Additional file 16); thus, the association measure, Spearman Correlation Coefficient, was left at default and createSWSFnetFromFile was used to create the network model with no other parameter settings. The model’s hub genes were identified by calculating the connectivity degree (number of neighbors) of each node (gene, transcript) using the function: getVertexStats.

### Gene annotation and functional enrichment of GO categories

Genes were annotated from three sources. *Arabidopsis* orthologs were identified using The Arabidopsis Information Resources (TAIR) annotation in Gramene [106]. Additional annotations were added from Grimplet et al. [107] and the corresponding author. A GO file was created using the EnsemblPlants BioMart [108] for *Vitis vinifera.* Functional category enrichment of biological processes was determined with the BinGO plugin application (version 3.0.2) in Cytoscape (version 3.2.1) [109]. Gene ontology membership classifies function hierarchically from broad to specific. Multiple testing correction adjusted *p*-values were determined using the Benjamini & Hochberg false discovery rate (FDR) at a 0.05 threshold.

## Additional files

Additional file 1: Read counts uniquely mapped to the PN40024 grape genome (XLSX 12905 kb).

Additional file 2: Log_2_ counts per million of filtered and normalized read counts (XLSX 11815 kb).

Additional file 3: Differential expression results from edgeR of the significantly affected transcripts in common from berry skins of seven grapevine cultivars at different °Brix levels. V1 ID refers to the transcript identification of the gene loci of the V1 version of the PN40024 *Vitis vinifera* reference genome. Cultivar abbreviations are Cabernet Sauvignon (CS), Merlot (ME), Pinot Noir (PN), Chardonnay (CD) and Semillon (SM). *P*-values and adjusted *p*-values (PAdj) were calculated for each of the comparisons between the different °Brix levels for each cultivar (XLSX 2391 kb).

Additional file 4: Annotation of the differentially expressed genes (DEGs) and count data associated with each gene. V1 ID is the same as in Additional file 3. AtID refers to *Arabidopsis thaliana gene loci*. Headings on the column refer to the cultivar abbreviation followed by the number of the replicate and the number of the °Brix level. The final columns compute the differences between °Brix levels for each cultivar and average them for all cultivars. Color highlights represent decreasing (green) or increasing (red) differences for the gene for the 26 to 20 °Brix comparison (26–20) (XLSX 3136 kb).

Additional file 5: Statistical results from gene set enrichment analysis of the DEGs using BinGO (XLSX 271 kb).

Additional file 6: Image of the network construction of the gene ontology categories by BinGO. Yellow colors represent significant enrichment and size of the circle represents the number of genes in each set (PDF 214 kb).

Additional file 7: BinGO analysis of the top 500 DEGs with a positive difference between 26 and 20° Brix (26–20) (XLSX 73 kb).

Additional file 8: BinGO analysis of the top 500 DEGs with a negative difference between 26 and 20° Brix (26–20) (XLSX 88 kb).

Additional file 9: A gene cluster dendrogram with gene modules (colored blocks) for the cuttreeDynamic and merged modules functions in WGCNA (PDF 11 kb).

Additional file 10: Scale independence and connectivity plots for the determination of the power function of a scale-free topology using the soft thresholding function in WGCNA (PDF 5 kb).

Additional file 11: Module membership of all filtered transcripts as defined by WGCNA. Values are the kME (module eigengene connectivity). Circadian rhythm and clock genes are highlighted in yellow (XLSX 17289 kb).

Additional file 12: Summary details of all 64 gene modules. Gene module rows are highlighted with different colors to reflect transcript abundance differences between red and white grapes for each module (red highlight for red grapes, green highlight for white grapes and yellow highlight for no distinction between red and white grapes) (XLSX 99 kb).

Additional file 13: Annotations of the top 100 hub (kME) genes in the turquoise module. Highlight colors represent different GO categories assigned to each gene (XLSX 111 kb).

Additional file 14: Network statistics for the petal analysis on the filtered set of 15,092 genes. V1 ID is as in Additional file 3. The table includes all genes of the network model based on a Spearman correlation threshold of 0.703. Each gene’s cluster coefficient (localCC) and connectivity degree (k) are included (XLSX 423 kb).

Additional file 15: The set of 216 genes in the hub module constructed from the top 5% of the most connected genes in the petal-constructed gene network. Column headings are the same as in previous additional files (XLSX 76 kb).

Additional file 16: Top) Histogram (blue) of the 16,606 transcripts and line (line) of expected normal distribution considering the mean and standard deviation of the given data. Bottom) Quantile-quantile (Q-Q) plot of given data compared to the expected hypothetical normal distribution. Histogram and Q-Q plots demonstrate that the distribution of the given data is not normal as the histogram lies outside the normal curve and the points on the QQ-plot are not scattered alone the red line (PDF 580 kb).

## Abbreviations

2 ATG11: AuTophaGy-related 11
ABA: Abscisic acid
APG9: AutoPhaGy 9
ARC5: Accumulation and Replication of Chloroplast 5
ARF3: Auxin Response Factor 3
ASR: Abscisic acid Stress Ripening
ATG18g: Homolog of yeast AuTophaGy 18 g
ATG2: AuTophaGy
BARD: United States – Israel Binational Agricultural Research and Development Fund
Bp: Base pair
CCA1: Circadian Clock Associated 1
CIP4: COP1 Interacting Protein 4
COL4: COnstans-Like 4
COP1: COnstitutive Photomorphogenic 1 CREs: cis-regulatory elements
CRY3: CRYptochrome 3
CURT1: CURvature Thylakoid 1
DDM1: Decrease in DNA Methylation 1
DEG(s): Differentially Expressed Gene(s)
EFS: Early Flowering in Short days
ELF: EarLy Flowering
EMF1: Embryonic Flower 1
FAR1: Far-Red impaired Response 1
FDR: False Discovery Rate
FLC: Flowering Locus C
FRS5: FAR1-Related Sequence 5
FT: Flowering locus T
GO: Gene Ontology
GPT2: Glucose Phosphate Transporter 2
HDA6: Histone DeAcetylase 6
HKT1: High-affinity K (potassium) Transporter 1
HMG: High Mobility Group
HSL1: HAESA-like 1
HUB: Histone Ubiquitin ligase
HXK1: HeXose Kinase
HY5: Elongated HYpocotyl 5
kME: Module Eigengene connectivity
LHY: Late elongated HYpocotyl
LNK2: Light-iNducible and clock-regulated 2
MAF4: MADS Affecting Flowering 4
MAF5: MADS Affecting Flowering 5
MBD9: Methyl-CPG-Binding Domain 9
MCM: MiniChromosome Maintenance
ME: Module Eigengene
MORC4: MicrORChidia 4
NCBI: National Center for Biotechnology Information
NPH3: Non-Phototropic Hypocotyly 3
OPR3: OxoPhytodienoate Reductase 3
PCA: Principal Component Analysis
PcG: Polycomb group
PCL1: PhytoCLock 1
PHYB: PHYtochrome B
PHYE: PHYtochrome E
PIE1: Photoperiod Independent Early Flowering 1
PPR: PentatricoPeptide Repeat
PRR9: Psuedo-Response Regulator 9
PTAC16: Plastid Transcriptionally Active 16
RCC1: Regulator of Chromosome Condensation
RVE1: ReVeillE 1
SKIP: SNW/SKI-Interacting Protein
SNF: Sucrose Non-Fermenting
SnRK1.1: SNF1 Related protein Kinase
SPA3: Suppressor of Phytochrome A-related 3
TAIR: The Arabidopsis Information Resource
TCP7: Transcription factor TCP domain protein 7
TF(s): Transcription Factor(s)
TIC: Time for Coffee
TOR: Target Of Rapamycin
TPL: ToPLess
Vvi: *Vitis vinifera*
WGCNA: Weighted Gene Co-expression Network Analysis
XCT: XAP5 Circadian Timekeeper
ZTL: ZeiTLupe
