# Supplementary figures and images for: The common transcriptional subnetworks of the grape berry skin in the late stages of ripening
Source: BMC Plant Biol. 2017 May 30;17:94. doi: 10.1186/s12870-017-1043-1 (PMC5450095; doi:10.1186/s12870-017-1043-1)

# BinGO constructed network of biological processes

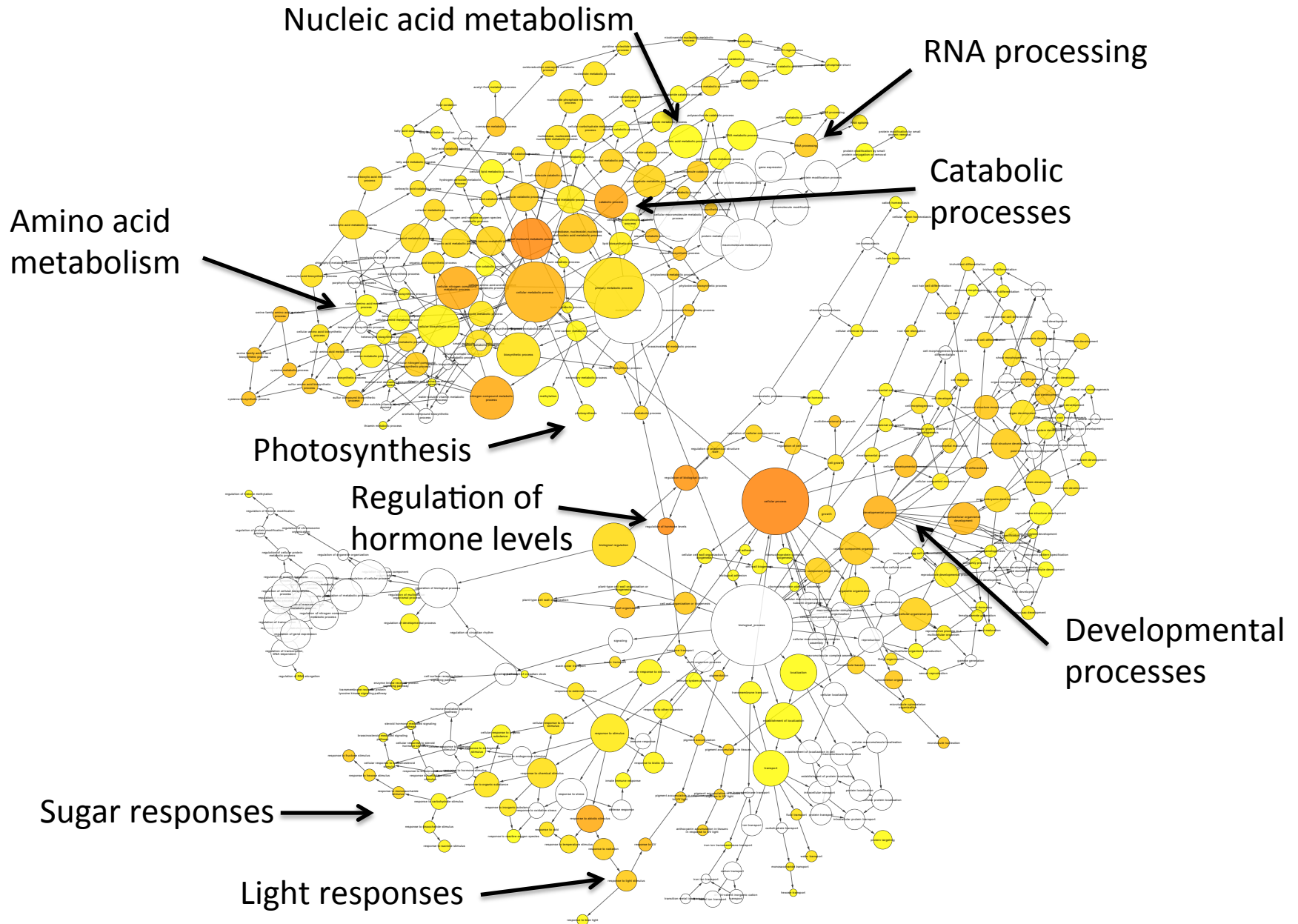

Supplement: Supplementary file 6 — Image of the network construction of the gene ontology categories by BinGO. Yellow colors represent significant enrichment and size of the circle represents the number of genes in each set (PDF 214 kb). [file 12870_2017_1043_MOESM6_ESM.pdf]

### Scale independence

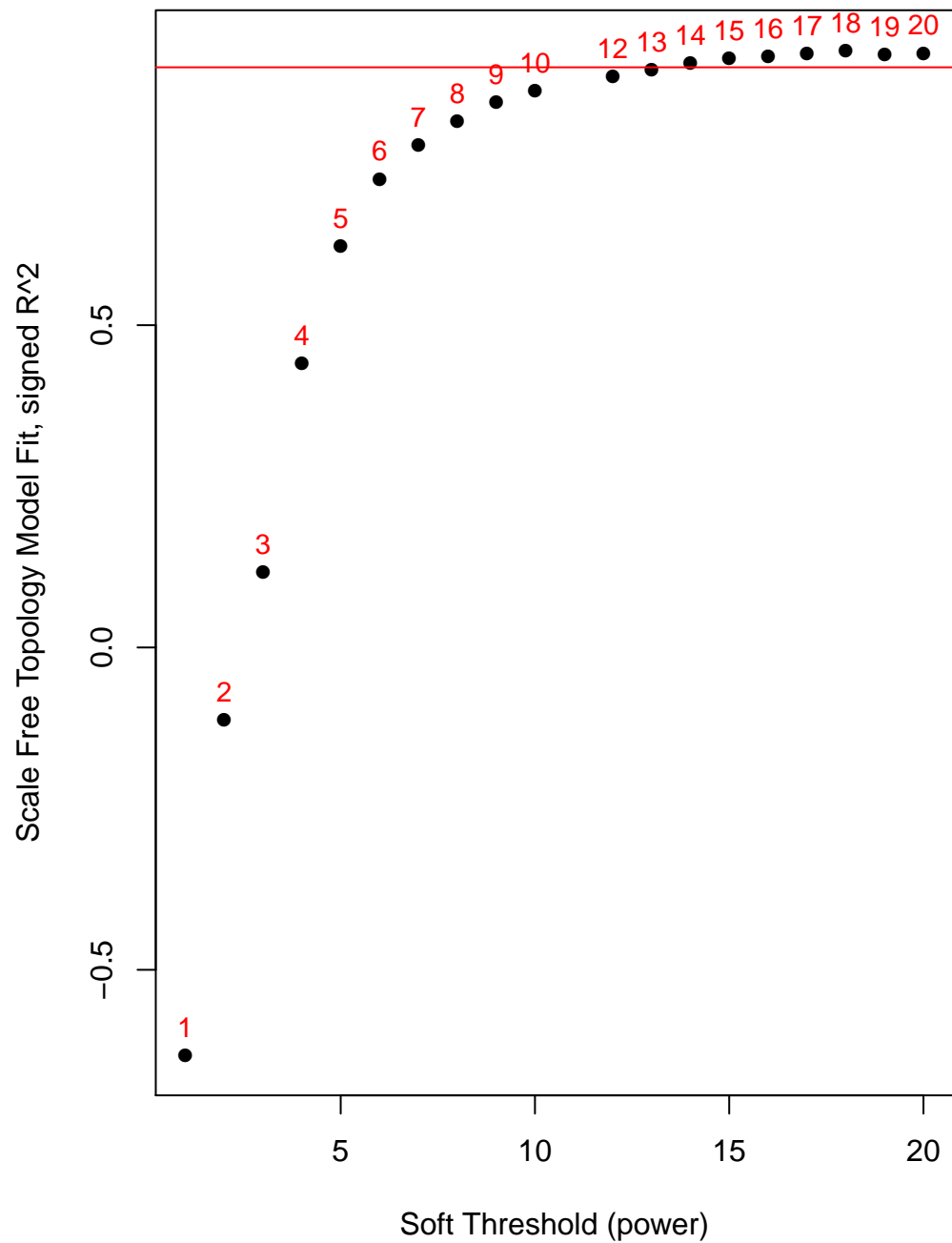

### Mean connectivity

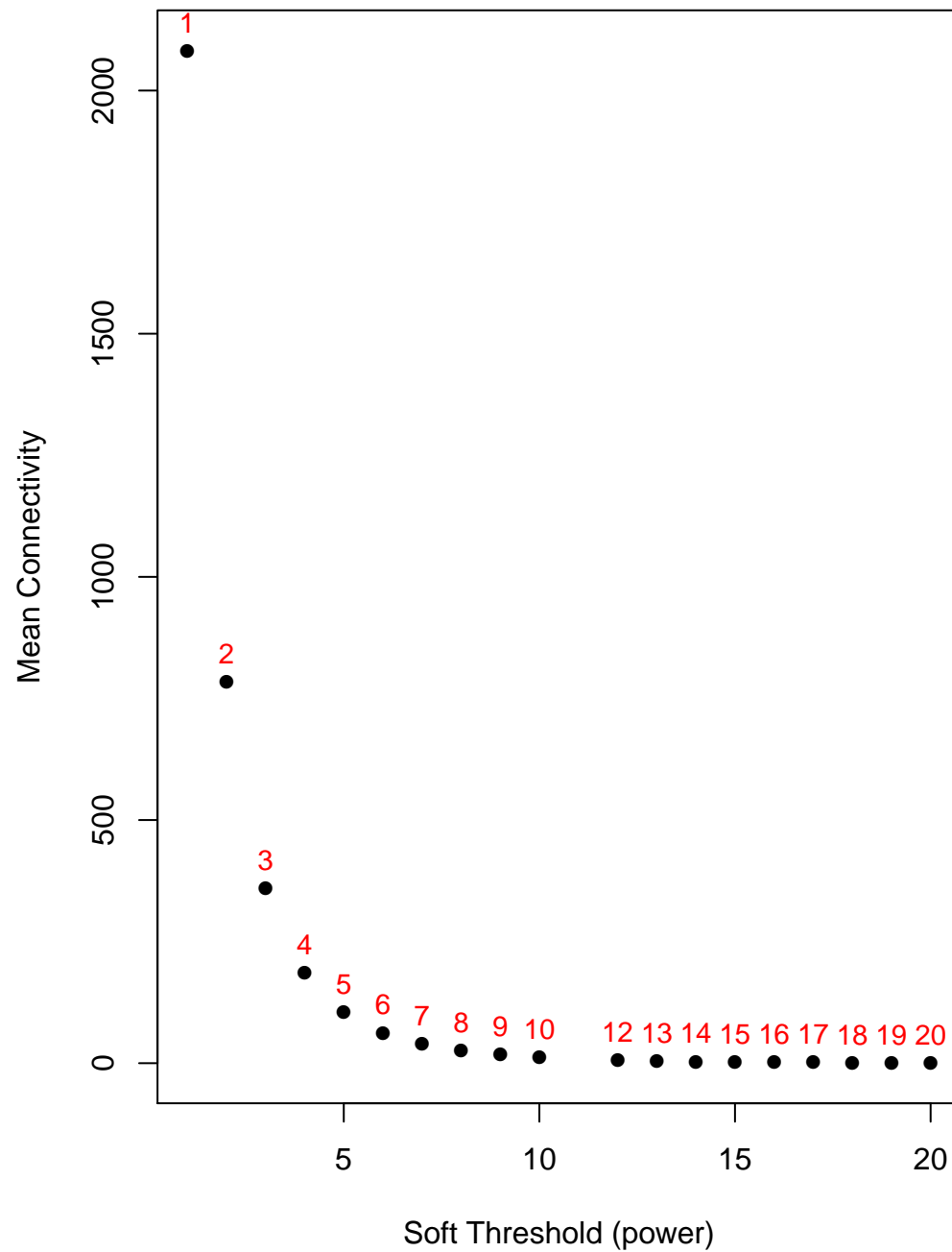

Supplement: Supplementary file 10 — Scale independence and connectivity plots for the determination of the power function of a scale-free topology using the soft thresholding function in WGCNA (PDF 5 kb). [file 12870_2017_1043_MOESM10_ESM.pdf]

**Histogram of Data**

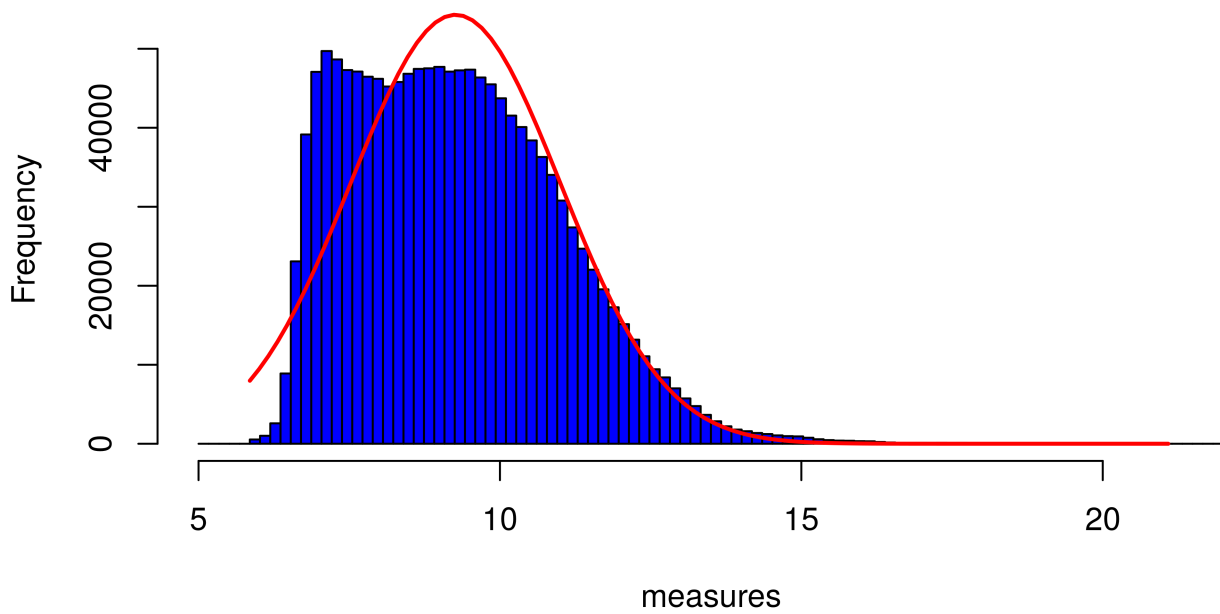

**Normal Q-Q Plot**

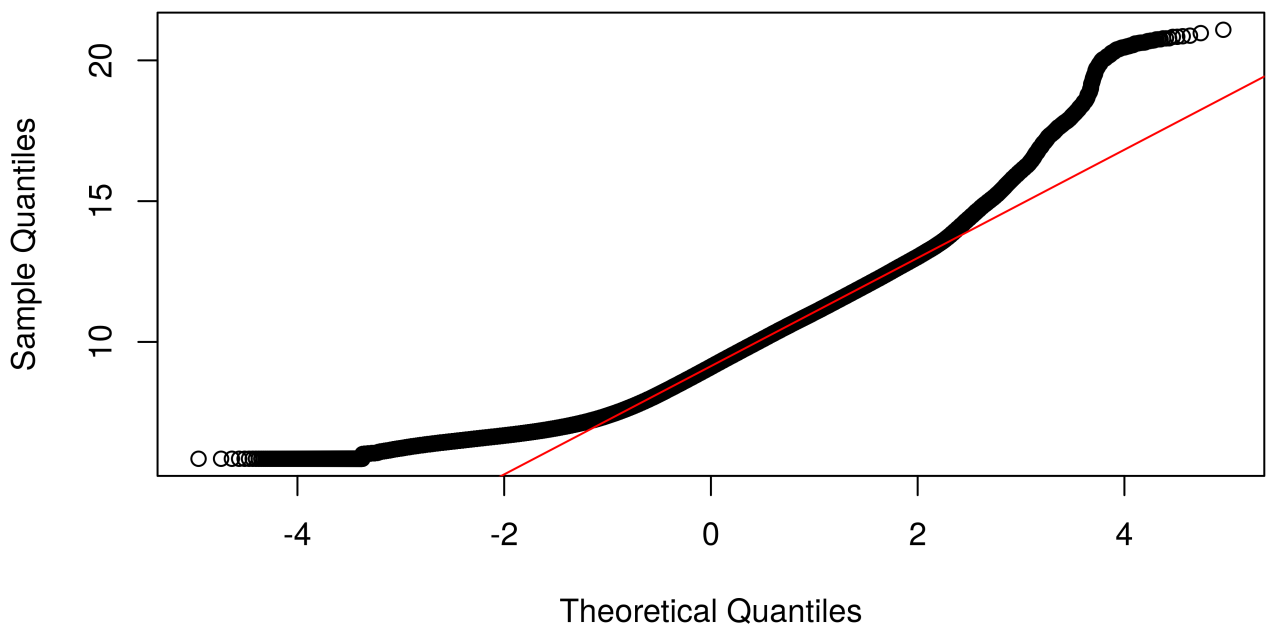

Supplement: Supplementary file 16 — Top) Histogram (blue) of the 16,606 transcripts and line (line) of expected normal distribution considering the mean and standard deviation of the given data. Bottom) Quantile-quantile (Q-Q) plot of given data compared to the expected hypothetical normal distribution. Histogram and Q-Q plots demonstrate that the distribution of the given data is not normal as the histogram lies outside the normal curve and the points on the QQ-plot are not scattered alone the red line (PDF 580 kb). [file 12870_2017_1043_MOESM16_ESM.pdf]
