# Supplementary material for: The common transcriptional subnetworks of the grape berry skin in the late stages of ripening
Source: BMC Plant Biol. 2017 May 30;17:94. doi: 10.1186/s12870-017-1043-1 (PMC5450095; doi:10.1186/s12870-017-1043-1)

Outlier dendrogram and trait heatmap

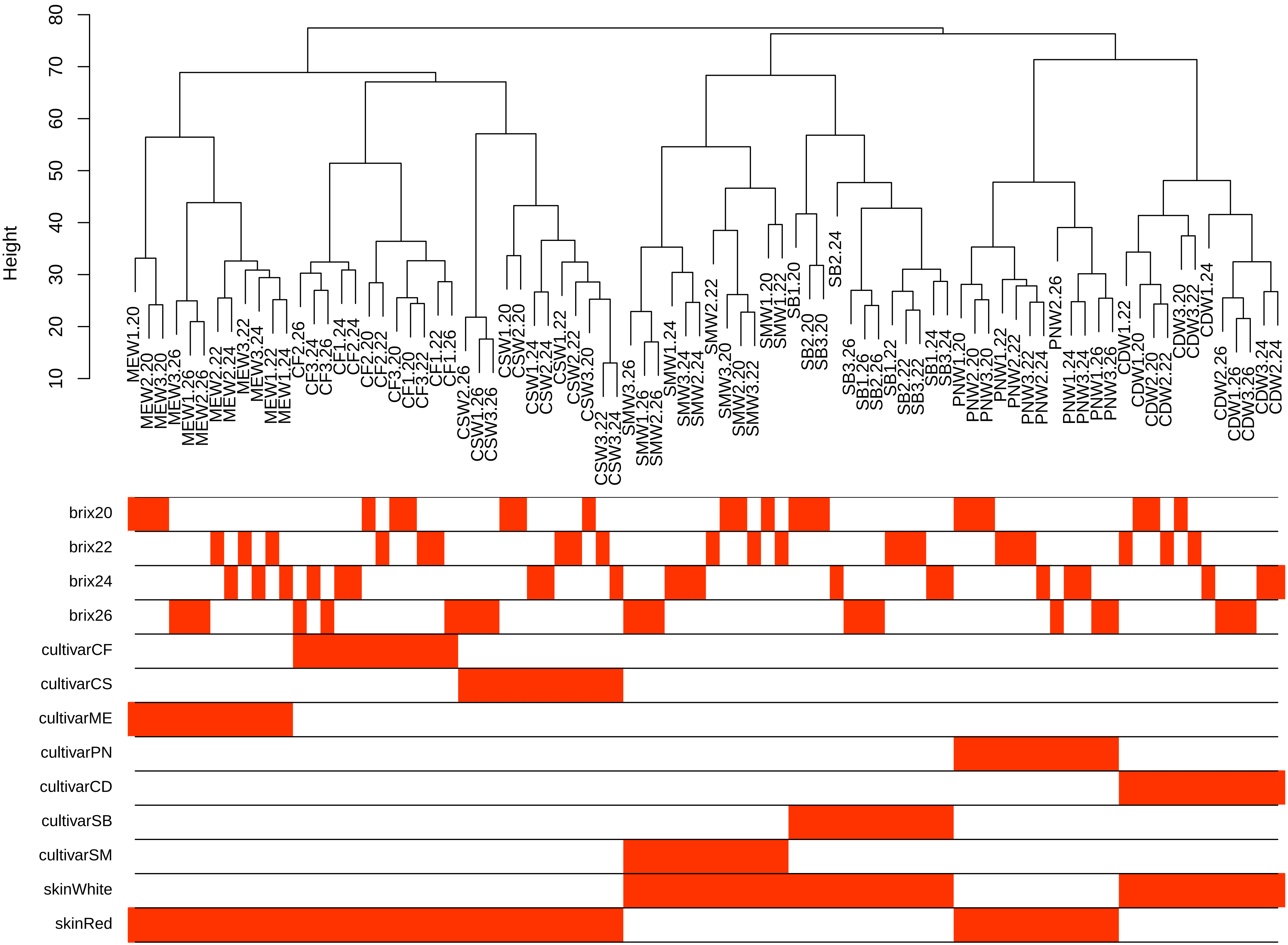

Supplement: Supplementary file 9 — A gene cluster dendrogram with gene modules (colored blocks) for the cuttreeDynamic and merged modules functions in WGCNA (PDF 11 kb). [file 12870_2017_1043_MOESM9_ESM.pdf]
